# Supplementary material for: Changing Epidemiological Trends of Hepatobiliary Carcinomas in Austria 2010–2018
Source: Cancers (Basel). 2022 Jun 23;14(13):3093. doi: 10.3390/cancers14133093 (PMC9264900; doi:10.3390/cancers14133093)
Supplement: Supplementary file 1 [file cancers-14-03093-s001.zip › cancers-1759275-supplementary.pdf]

cancers

Supplementary Materials

Changing Epidemiological Trends of Hepatobiliary Carcinomas in Austria 2010–2018

Florian Hucke [1,*], Matthias Pinter [2,3], Miriam Hucke [1], Simona Bota [1], Dajana Bolf [1], Monika Hackl [4] and Markus Peck-Radosavljevic [1]

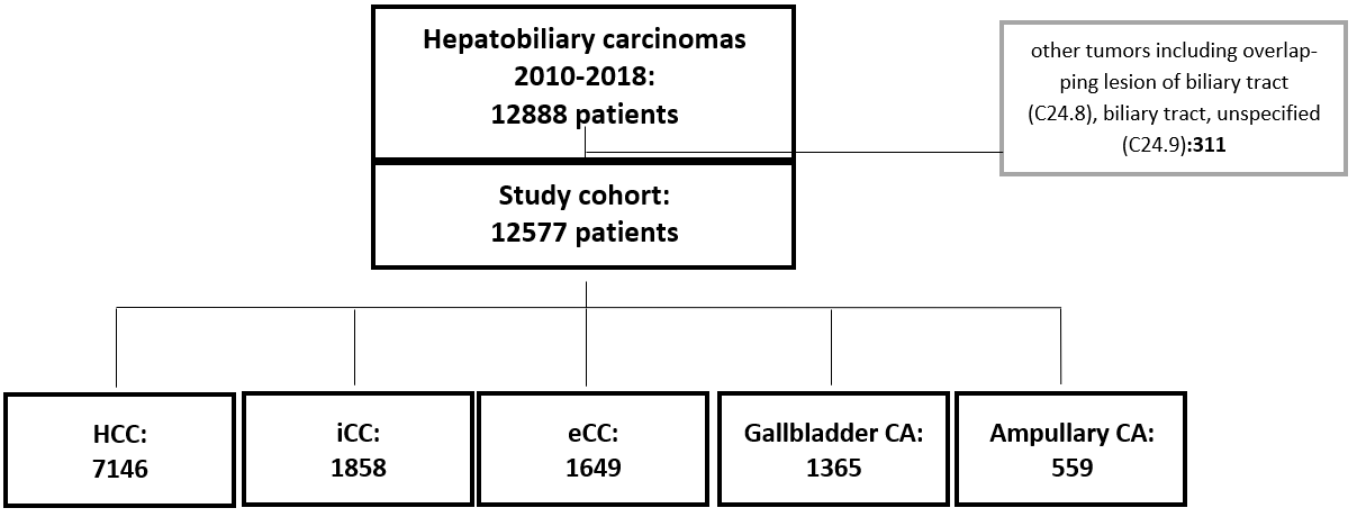

Figure S1. Flowchart diagram of patient inclusion and exclusion. Abbreviations: HCC, hepatocellular carcinoma; iCC, intrahepatic cholangiocarcinoma; eCC, extrahepatic cholangiocarcinoma; CA, carcinoma.
